# Castor Oil and Spirits of Turpentine in Typhiod Fever

**Published:** 1849-06

**Authors:** 


### CASTOR OIL AND SPIRITS OF TURPENTINE IN TYPHOID FEVER.

Dr. C. W. Crozier, of Russellville, Ky., has communicated to us the particulars of a case of typhoid fever in which, at an advanced period, castor oil and spirits of turpentine were administered with success. The life of the patient had been despaired of by her friends, and her recovery, Dr. C. attributes to the use of these remedies, in doses of a teaspoonful each, repeated three times daily. While employing this mixture, he had the patient bathed in tepid ley, the effect of which seemed to be salutary. This practice in typhoid fever, attended with coma and watery discharges from the bowels, was suggested to Dr. C. by Dr. James H. Baker, of Knox county, Tenn., in whose hands Dr. C. says it has been found valuable.

